# Progress in Surgery

**Published:** 1898-02-12

**Authors:** 


### PROGRESS IN SURGERY.

### SURGERY OF THE LIVER AND GALL-BLADDER.

(*Continued from page* 332.)

**Cholecystotomy**, or cholecystostomy, is the operation *par excellence* in the treatment of gall-stones, says Robson.[18] Its indications are: (1) In all cases where the gall-bladder is sufficiently large to permit of drainage, after gall-stones have been removed from the gall-bladder or ducts; (2) in cases where there are gall-stones in the ducts, but the patient is too ill to bear a prolonged operation, the gall-stones being deliberately left for treatment by some solvent solution; (3) in empyæma of the gall-bladder where that viscus is not too much disorganised to be permitted to remain; (4) in certain cases of chronic catarrh of the gall-bladder or bile-ducts; (5) in suppurative cholangitis; (6) in obstruction of the ducts due to hydatid disease; (7) in dropsy of the gall-bladder; (8) in idiopathic rupture, or laceration, or gun-shot injury of the gall-bladder or ducts; (9) in cases of choledochotomy in order to avoid tension in the sutured duct; (10) in certain cases of obstructive jaundice dependent on malignant tumour which is occluding the ducts, but in these cases the increased danger must be borne in mind; and (11) in some cases of phlegmonous cholecystitis, or gangrene, where the patient is too ill to bear cholecystectomy. Fuchs[19] states that Mikulicz successfully treated a case of perforation of the gall-bladder, following on an acute cholecystitis, by cholecystostomy and drainage; and Michaux[20] has similarly treated a case of infective angiocholecystitis without lithiasis.

**Calculi in the common bile-duct** occur only in about 4 per cent. of all cases, and are often associated with atrophy of the gall-bladder. Robson[21] gives the following methods of treatment: (1) In a few cases a concretion of moderate size may be manipulated backwards into the cystic duct, and thence extracted by scoop or forceps; but this is seldom practicable on account of the contraction of the gall-bladder and cystic duct; (2) occasionally a small stone may be pressed into the duodenum, but this is rare, and not infrequently it may be pushed into a dilated diverticulum of Vater, and so be missed, and the whole operation rendered futile; (3) cholecystostomy, with subsequent treatment by solvent injections, is well worth bearing in mind, on account of its simplicity and safety, together with the certainty of giving immediate relief with a modicum of risk, and putting the patient in better condition for subsequent treatment should such be necessary; (4) cholelithotrity, or crushing the stones *in situ*; (5) needling concretions through the duct-walls; (6) choledochotomy, or incising the duct and removing the calculi; (7) choledocho-duodenotomy, or reaching the duct through the opened duodenum for stones impacted in the duodenal end of the duct; (8) cholecystenterostomy, or short-circuiting the obstruction; (9) choledochoenterostomy, or uniting the duct to the gut in case of largely dilated ducts; and (10) choledochostomy, or attaching the dilated duct to the surface and draining it. The author thinks there is no danger if the finger and thumb only be used as the compressing force for crushing the stones. The best means of dissolving the calculi is to syringe a warm ·5 per cent. solution of sapo animalis or warm olive oil through the fistula night and morning.

**Cholecystectomy**, or excision of the gall-bladder, may, according to the same author,[22] be required: (1) In bullet wound or other wound of the gall-bladder where suture is impracticable; (2) in phlegmonous cholecystitis; (3) in gangrene of the gall-bladder; (4) in multiple or in perforating ulcers; (5) in chronic cholecystitis from gallstone, where the gall-bladder is shrunken and too small to drain safely and where the common duct is free from obstruction; (6) in mucous fistula due to stricture of the cystic duct; (7) in hydrops of the gall-bladder due to stricture of the cystic duct, as also in certain cases where the gall-bladder is very much dilated; (8) in certain cases of empyæma where the walls of the gall-bladder are very seriously damaged; and (9) in cancer of the gall-bladder.

**Cholecystenterostomy** is indicated[23]: (1) In biliary fistulæ, depending on stricture or other permanent occlusion of the common duct; (2) very occasionally in cancer of the head of the pancreas or malignant tumour of the common duct, leading to chronic jaundice and distended gall-bladder; but in such cases the mortality is necessarily so high that the justifiability of the operation is questionable. (3) occasionally in impaction of gall-stones in the ducts, where the patient is not in a fit condition to bear the more prolonged operation of separating adhesions, and crushing or removing the concretions by choledochotomy; and (4) in certain cases of obstruction of the cystic duct where cholecystectomy is impracticable. The operation is contra-indicated: (1) In any obstruction of the bile-ducts which can be cleared away with reasonable probability of success; (2) in malignan disease of the head of the pancreas or common bile-duct, leading to distension of the gall-bladder, the mortality is so great (eight operations with seven deaths) that it is hardly worth doing; (3) in contracted gall-bladder, where it is impracticable to insert the button; (4) where there are extensive adhesions which would lead to kinking of the bowel; and (5) in very large gall-bladder with obstruction of the cystic duct, where cholecystectomy should be done. A fatal case, associated with malignant disease, in which the anastomosis was performed by means of Murphy's button, is reported by Scott.[24]

**Fistula of Gall-bladder.**—After cholecystotomy, says Robson,[25] this does not occur if the edge of the gall-bladder incision is sutured to the aponeurosis, and not to the skin. He divides fistula[26] into those resulting from operation and those from disease (gall-stones or cancer). Biliary cutaneous fistulæ are most common. If pathological they point at the umbilicus. The post-operative forms are either mucous or biliary. The mucous form discharges only about an ounce of fluid daily and causes little inconvenience; the biliary form discharges large quantities of bile, and interferes with the health and comfort of the patient. If the fistula is small, the application of the actual cautery is advised. This failing, the abdomen may be opened, the gall-bladder detached, and the opening sutured, or the fistula may be dissected from its cutaneous margin, the orifice of the fistula being then closed accurately with sutures.

**Tumours of the Gall-bladder** are classified[27] as follows: (1) Those caused by distension of bile, concretions, pus (empyema), or mucus (hydrops); and (2) those caused by new growths, benign and malignant. Those tumours due to distension of the gall-bladder by concretion or bile are very rare, while those of the other varieties occur more frequently. The diagnosis must be made from: (1) movable right kidney; (2) tumour of the right kidney or of the suprarenal capsule; (3) tumour of the intestine or fæcal impaction: (4) tumour of the liver; (5) pyloric tumour; and (6) abnormal projection of the liver. Innocent growths of the gall-bladder are rare, as are also the malignant, when primary. The malignant tumours include the columnar-celled carcinoma, the spheroidal-celled carcinoma, sarcoma, and squamous epithelioma. Secondary malignant tumours are associated most frequently with gall-stones (Zinkert gives 85 per cent.), and are usually diagnosed by their rapid growth and characteristic hardness. Tumours of the bile-ducts are classified similarly to those of the gall-bladder. The diagnosis of tumours of the gall-bladder from impacted gall-stones is practically impossible.

**Infective and Suppurative Cholangitis**, or suppurative catarrh of the bile-ducts, is caused[18] by cholelithiasis, hydatid disease, malignant disease of the liver, and typhoid fever. By means of cholecystostomy for this affection, Terrier says, we get: (1) The evacuation of the septic contents of the gall-bladder; (2) calculi, which are most frequently present there, are removed; (3) the other biliary passages, more or less obstructed, either by calculi or by swelling of their walls, are rendered as free as possible; (4) the septic bile is allowed to escape, and mechanically washes out the lower passages, carrying away through the drainage-tube many of the infectious elements; (5) the relief of pressure prevents absorption of the septic elements; and (6) the relief to the kidneys by allowing the bile to escape more freely is also of importance, as they are thus enabled to perform their function more freely in relieving the system of septic and other materials. Terrier employs drainage until a bacteriological examination of the discharge shows it to be sterile, or nearly so.

**Pyloric Obstruction of Hepatic Origin** as a sequel of cholelithiasis is reported in two cases by Tuffier and Marchais.[29] If washing out the stomach fails, adhesions must be separated, but should this be impossible or too risky, a gastro-enterostomy, which affords the best means of relieving the symptoms, is to be performed.

[18] Lancet, June 19, 1897, p. 1,667. [19] Brit. Med. Journ., Aug. 28, 1897. [20] Med. Week, Ap. 30, 1897. [21] Lancet, June 19, 1897, p. 1,669. [22] Ibidem, p. 1,670. [23] Ibidem, p. 1,671. [24] Australas. Med. Gaz., June 22, 1897, p. 275. [25] Lancet, June 19, 1897, p. 1,663. [26] Lancet, June 12, 1897; and Med. Mag., June, 1897. [27] Ibidem. [28] Lancet, May 29, 1897, p. 1,454. [29] New York Med. Rec., July 17, 1897; and Rev. de Chir. de Paris, 1897.

